# Inverse Statistics of Active Matter Trajectories to Distinguish Interaction Kernel Anisotropy from Emergent Correlations

**DOI:** 10.1007/s11538-026-01668-6

**Published:** 2026-06-07

**Authors:** Simon F. Martina-Perez

**Affiliations:** School of Medicine and Biomedical Sciences, University of Oxford, Oxford, UK

**Keywords:** Interacting particle systems, Statistical mechanics, Fokker-Planck Equation, Trajectory analysis

## Abstract

High-resolution imaging provides dense trajectories of migrating cells, flocking animals, and synthetic active particles, from which interaction laws can be determined with a wide variety of methods. Yet, distinguishing whether front-back or lateral biases seen in such data reflect intrinsic anisotropy in the interaction kernel or emergent correlations that are nevertheless produced by isotropic pairwise interaction forces remains an open challenge. We resolve this ambiguity by deriving a linear partial differential equation that connects measurable two-point velocity correlations to an unknown, distance- and angle-dependent interaction kernel. Turing-like instabilities can occur which allows for dipolar or quadrupolar patterns to arise even when agents interact according to an underlying attraction-repulsion law that is angle-independent. We then show that incorporating a weak velocity-alignment force can interfere with anisotropic pattern formation by suppressing dipolar patterns. We validate these predictions with agent-based simulations and provide design guidance for experiments that seek to discriminate intrinsic anisotropy from emergent effects.

## Introduction

Collective motion of individual agents in living and artificial systems is often captured by models in which self-propelled particles (SPP) interact through pairwise forces that depend only on their mutual distance (Vicsek et al. 1995; Couzin et al. 2002; D’Orsogna et al. 2006; Szabó et al. 2006). Despite their simplicity, canonical SPP models recapitulate a wide repertoire of emergent patterns that are ubiquitous in nature: flocking, milling, phase-separated clustering and emergent ordering (Vicsek et al. 1995; D’Orsogna et al. 2006). All of these structures have clear biological analogues: rotating mills of fish, coherent flocks of birds, and the collective streaming observed in epithelial sheets during wound healing (Couzin et al. 2002; Szabó et al. 2006). The phenomenological success of these models has long motivated the inverse problem: inferring the underlying interaction laws directly from experimentally observed trajectory data with the aim of uncovering their mechanistic basis. One question of particular interest is determining whether agents exchange information in a direction-dependent (anisotropic) manner (Huth and Wissel 1992; Couzin et al. 2005; Herbert-Read et al. 2011; Francisco et al. 2019; Rosenthal et al. 2015).

A common strategy to detect anisotropy in the rules governing interactions between agents is to compute two-point spatial or velocity correlations and test whether the field is isotropic. Bayesian model comparison of such statistics has been used to quantify directional bias with minimal modelling assumptions (Giuggioli et al. 2015; Jiang et al. 2017). Further, mapping the locations of cell-cell contacts on the cell membrane and mapping subsequent velocity changes has shown how electrotactic stimuli disrupt the spatial patterns of cell-cell interactions among human corneal epithelial cells (Crossley and Martina-Perez 2025). Correlation methods are appealing for high-throughput imaging assays, but they do not by themselves reveal the underlying forces driving any observed anisotropy. Over the last decade a range of statistical and machine-learning tools have been developed for this task. Rather than review all such methods, we highlight a few representative methods. Sparse identification of nonlinear dynamics (SINDy) (Brunton et al. 2016) and its weak-form variant WSINDy have been extended to second-order agent systems; a recent study used WSINDy to learn anisotropic interaction laws in heterogeneous cell monolayers and automatically clustered cells into behavioural subtypes (Messenger et al. 2022). Non-parametric regression frameworks couple consistency theorems with efficient algorithms to recover distance-based kernels from many trajectories (Fei et al. 2019, 2021). Together these approaches provide interpretable force laws, but they often incorporate angular basis functions by default, implicitly assuming that any directional bias visible in the data reflects a directional force. In contrast to methods that explicitly parameterise interaction kernels using angular basis functions, attention-based neural-network approaches do not *a priori* impose anisotropy. Instead, directional interaction patterns may emerge implicitly through learned attention weights that depend on the relative configuration of neighbouring agents, allowing the model to capture pairwise and higher-order relationships directly from trajectory data. Such deep learning based attention network approaches have recently been applied to infer directional interaction rules in both animal and cellular systems: for example, deep attention networks uncovered the anisotropic social forces guiding zebrafish shoals (Heras et al. 2019), and analogous architectures have been used to learn direction-dependent cues in collective cell migration (LaChance et al. 2022). Similar deep attention frameworks have also facilitated the identification of invasion and migration genes in neural crest and melanoma cells from high-throughput screens (Kulesa-Kasemeier et al. 2025).

This assumption is problematic, because, as will be shown in this work, anisotropic correlations can emerge even when the true interaction kernel is strictly isotropic. Sampling artefacts and uneven neighbour distributions can induce spurious angular structure even when the true kernel is isotropic. Spurious detection of anisotropic patterns in truly isotropic force fields is not confined to kinetically interacting agents. For instance, in seismic surface-wave tomography (a geological application) uneven ray coverage around sharp velocity contrasts produces spurious angular anisotropy, despite the Earth’s material being isotropic (Paul et al. 2024). Incorporating these artefacts as genuine directional forces not only over-complicates the model but also masks the true driving mechanisms. Hence, anisotropic interactions should be introduced only when the data provide clear evidence for them. This work uses a kinetic theory-based analysis to determine under what conditions two-point velocity correlations reliably reflect intrinsic directional biases in an otherwise isotropic SPP framework. The main findings are that observing anisotropic statistics does not, by itself, justify baking anisotropy into the governing equations. Instead, one must first rule out alternative origins of apparent directionality, such as persistence, inertial delays, or nonlinear density pattern formation, before attributing it to anisotropic forces.

The structure of this work is as follows. Starting from a canonical SPP model, we derive a closed Fokker-Planck equation for the two-particle velocity probability density, and a Kramers-Moyal expansion (Risken and Frank 1996; Kramers 1940; Moyal 1949) is used to find the governing equation for the spatial correlation field in Section 2. Linear stability analysis of the resulting partial differential equation (PDE) in Section 3 shows that front-back or lateral biases can emerge in this correlation even though the interaction kernel is angle-independent, and that dipolar interaction kernels can suppress a dipolar instability in the emergent correlation pattern. Finally, in Section 4, in addition to isotropic attraction-repulsion kernels, we also incorporate an alignment term and show that it can selectively shift the dispersion relations of some of the unstable correlation modes, allowing us to probe how alignment suppresses or enhances angular instabilities.

## Governing Equations of Velocity Correlations

The purpose of this section is to derive a closed-form PDE for the velocity-velocity correlation field of two interacting particles. In Section 2.1 the Fokker-Planck equation describing the probability density for the velocities of two interacting particles is derived. Then, in Section 2.2 a closed-form expression for a correlation function in the frame of motion of a single particle is found by using the Fokker-Planck equation. This is used in Sections 3.1-3.3, which investigate the conditions under which anisotropy can arise in the correlation function, and show that this can occur with a simple, isotropic interaction kernel.

### Pair-Density Formulation

This work considers the interactions between *N* identical particles with positions ${\textbf {x}}_i(t)$ and velocities ${\textbf {v}}_i(t)$ that obey the following dynamics,

1 $$\begin{aligned} \dot{{\textbf {x}}}_i&= {\textbf {v}}_i, \end{aligned}$$

2 $$\begin{aligned} \dot{{\textbf {v}}}_i&= S({\textbf {v}}_i)\,{\textbf {v}}_i + \frac{1}{N}\sum _{j\ne i} f_{ar}(r_{ij}, \phi _{ij})\,\hat{{\textbf {r}}}_{ij} + \sigma \,\boldsymbol{\xi }_i(t). \end{aligned}$$

Here, $r_{ij}= \Vert {\textbf {x}}_j-{\textbf {x}}_i\Vert$, $\hat{{\textbf {r}}}_{ij}= ({\textbf {x}}_j-{\textbf {x}}_i)/r_{ij}$, and and $\phi _{ij}$ is the angle between ${\textbf {v}}_i$ and ${\textbf {x}}_j - {\textbf {x}}_i$. The scalar self-propulsion term $S({\textbf {v}}_i)$ drives each particle toward a preferred speed and can be chosen to recover, for example, the persistent-random-walk of migrating cells (Szabó et al. 2006). The model allows the pairwise attraction-repulsion force, $f_{ar}(r_{ij},\phi _{ij})$, to depend on the separation vector, $\phi _{ij}$, and thus be anisotropic. Nevertheless, a commonly made assumption is that of an isotropic interaction force, reflecting the fact that many cells or animals sense neighbours primarily through distance cues such as adhesive contacts, chemotactic gradients or visual range. Because the interaction kernel, $f_{ar}(r,\phi )$ is left general, the framework that will be developed encompasses a wide class of pairwise interaction laws commonly used in collective motion, including Morse type attraction–repulsion forces, Lennard–Jones–type potentials, and other radial interaction kernels. Finally, $\boldsymbol{\xi }_i$ is standard Gaussian white noise with amplitude $\sigma$. The pair probability density, *P*, is given by

3 $$\begin{aligned} P({\textbf {r}},{\textbf {v}},{\textbf {v}}',t) = \left\langle \sum _{i\ne j} \delta \bigl ({\textbf {r}}-{\textbf {x}}_j+{\textbf {x}}_i\bigr )\, \delta \bigl ({\textbf {v}}-{\textbf {v}}_i\bigr )\, \delta \bigl ({\textbf {v}}'-{\textbf {v}}_j\bigr ) \right\rangle . \end{aligned}$$

Standard BBGKY reduction combined with a mean-field closure (also called molecular-chaos closure) shows that the pair probability density, *P*, obeys the Fokker-Planck equation  (Cercignani 1969), which is given by

4 $$\begin{aligned} \partial _t P + ({\textbf {v}}'-{\textbf {v}})\!\cdot \!\nabla _{{\textbf {r}}} P + (\mathcal {L}_{{\textbf {v}}}+\mathcal {L}_{{\textbf {v}}'})[P] = \frac{\sigma ^2}{2}\bigl ( \Delta _{{\textbf {v}}} + \Delta _{{\textbf {v}}'} \bigr ) P , \end{aligned}$$

where the operator $\mathcal {L}_{{\textbf {v}}}[P]$ groups together the deterministic self-propulsion drift and the mean-field attraction-repulsion term. See Appendix A for the derivation of the Fokker-Planck equation and more details.

### Velocity-Velocity Correlation Field

Given that particles move at near-constant velocity owing to the forcing term, *S*, the pair density, *P*, can be projected onto a fixed speed, $v=v'=v_0$, thus eliminating the fast radial dynamics of the speeds and focusing solely on the slower angular coordinates:

5 $$\begin{aligned} \tilde{P}(r,\varphi ,\varphi ',t) := \int _0^\infty \!\!\int _0^\infty P(r,{\textbf {v}},{\textbf {v}}',t)\, \delta (v-v_0)\delta (v'-v_0)\,v\,v'\,dv\,dv'. \end{aligned}$$

All angular integrations below refer to $\tilde{P}$. In the co-moving polar frame of a focal particle, *i*, with $\theta =0$ directed along its instantaneous velocity, the equal-time velocity-velocity correlation, *C*, is defined by

6 $$\begin{aligned} C(r,\theta ,t) = \Bigl \langle \hat{{\textbf {v}}}_i(t)\!\cdot \!\hat{{\textbf {v}}}_j(t)\; \Bigr |\; r_{ij}=r,\;\phi _{ij}=\theta \Bigr \rangle . \end{aligned}$$

Multiplying the Fokker-Planck equation (4) by $\hat{{\textbf {v}}}\!\cdot \!\hat{{\textbf {v}}}'$ and integrating over angular variables yields,

7 $$\begin{aligned} \partial _t C&= -v_0\,\partial _r C + D_r\bigl (\partial _r^2 + r^{-1}\partial _r\bigr )C + \frac{D_\theta }{r^2}\partial _\theta ^2 C - 2\gamma \,C + S_{\textrm{ar}}(r,\theta ), \end{aligned}$$

8 $$\begin{aligned} S_{\textrm{ar}}(r,\theta )&= \rho \, g(r,\theta )\, f_{\textrm{ar}}(r,\theta ), \end{aligned}$$

where $g(r,\theta )$ is the neighbour distribution around a focal particle, and $\rho$ is the global number density. The coefficients $v_0,\,\gamma =\sigma ^2/2v_0^2,\,D_r,\,D_\theta$ are single-particle kinetic statistics, representing, respectively: the mean self-propulsion speed; the angular decorrelation rate; the radial diffusivity of the pair separation; and the angular diffusivity of the separation vector. For clarity of exposition, we omit the derivation of Equation (7) here, see Appendix B for the derivation and further details.

## Emergence of Correlated Dipoles and Quadrupoles

The linear dynamics of the correlation field, *C*, are governed by fluctuations of the pair density projected at constant speed, which, when writing $\theta =\arg {\textbf {v}}$ and $\theta '=\arg {\textbf {v}}'$ for headings, and $\varphi =\arg {\textbf {r}}$ for the polar angle of the separation vector, is given by

9 $$\begin{aligned} \tilde{P}(r,\theta ,\theta ',\varphi ,t) :=\!\int _{0}^{\infty }\!\!\int _{0}^{\infty } P\!\bigl (r,{\textbf {v}},{\textbf {v}}',t\bigr )\, \delta (v-v_{0})\,\delta (v'-v_{0})\,v\,v'\,dv\,dv' . \end{aligned}$$

At this point, it is important to notice that inspection of

10 $$\begin{aligned} C(r,\Theta ,t)= \frac{\displaystyle \iint \cos (\theta -\theta ')\, \tilde{P}(r,\theta ,\theta ',\theta +\Theta ,t)\,d\theta \,d\theta '}{\displaystyle \iint \tilde{P}(r,\theta ,\theta ',\theta +\Theta ,t)\,d\theta \,d\theta '}, \end{aligned}$$

shows that any non-isotropic perturbation of $\tilde{P}$ feeds directly into the rise of angular structure in *C*. Linear stability is therefore most naturally performed on $\tilde{P}$ itself.

### Linear Stability of Pair Correlations

Projection of the Fokker-Planck pair equation onto $v=v'=v_{0}$, together with $\hat{{\textbf {v}}}=(\cos \theta ,\sin \theta )$ and $\hat{{\textbf {v}}}'=(\cos \theta ',\sin \theta ')$, yields the governing equation for the projected pair distribution, $\tilde{P}$,

11 $$\begin{aligned}&\partial _t \tilde{P} + \frac{v_0}{r}\Bigl [ \sin (\theta '-\varphi )\,\partial _\theta \tilde{P} - \sin (\theta -\varphi )\,\partial _{\theta '}\tilde{P} \Bigr ] - v_0\cos (\theta '-\varphi )\,\partial _r\tilde{P}\nonumber \\&\qquad = \widehat{\mathcal {L}}_r\tilde{P} + D_\theta \bigl ( \partial _{\theta \theta } + \partial _{\theta '\theta '} \bigr )\tilde{P}, \end{aligned}$$

where $D_\theta = \frac{\sigma ^{2}}{2}$, $\widehat{\mathcal {L}}_r\tilde{P} := -\partial _r\!\bigl [F(r)\,\tilde{P}\bigr ]$, and *F*(*r*) is an isotropic attraction-repulsion kernel. For a spatially homogeneous suspension, the stationary solution is

$$ \tilde{P}_{0}(r,\theta ,\theta ')=\frac{\rho _{0}}{(2\pi )^{2}}, $$

where $\rho _{0}$ is the bulk number density. Here, $\tilde{P}_{0}$ is independent of all angular and radial variables and normalised so that $\int \tilde{P}_{0}\,d\theta \,d\theta '=1$. The subsequent linear stability analysis will investigate the growth of angular modes in $\tilde{P}$. The existence of instabilities of such Fourier blocks maps one-to-one onto the emergence of front-back patterns in $C$. Write $\tilde{P}=\tilde{P}_{0}+\varepsilon \,\delta \tilde{P}$ and expand the perturbation in angular Fourier modes and the angularly averaged radial basis, *i.e.*, take the Hankel transform,

$$\begin{aligned} \delta \tilde{P}(r,\theta ,\theta ',t) =\sum _{m,n\in \mathbb {Z}}\int _0^\infty k\,\widehat{P}_{mn}(k,t)\,J_0(kr)\,e^{im\theta }e^{in\theta '}\,\text {d}k, \end{aligned}$$

Note that the Hankel transform is equivalent to averaging the plane wave $e^{ik r\cos \varphi }$ over its dominant direction to obtain $J_0$. Since $P_0$ is constant, perturbations obey

12 $$\begin{aligned} \partial _t \delta \tilde{P} =\,&\mathcal {L}_r\,\delta \tilde{P} + D_\theta (\partial _{\theta \theta }+\partial _{\theta '\theta '})\delta \tilde{P} - v_0\cos (\theta '-\varphi )\,\partial _r\delta \tilde{P}\nonumber \\&+\frac{v_0}{r}\big [\sin (\theta '-\varphi )\,\partial _\theta -\sin (\theta -\varphi )\,\partial _{\theta '}\big ]\delta \tilde{P}. \end{aligned}$$

Multiplying Equation (12) by $e^{-im\theta }e^{-in\theta '}$ and integrating over $\theta , \theta '$ allows to produce the following evolution equations. First, using that $\sin (\bullet -\varphi )=(1/2i)\big (e^{i(\bullet -\varphi )}-e^{-i(\bullet -\varphi )}\big )$ and $\partial _\bullet e^{im\bullet }=im\,e^{im\bullet }$ one obtains that upon projecting Equation (12) the following terms map onto nearest-neighbour couplings between angular modes,

$$\begin{aligned} \frac{v_0}{r}\sin (\theta '-\varphi )\,\partial _\theta \;&\leadsto \; \frac{v_0}{2r}\,m\big (\widehat{P}_{m,n-1}e^{+i\varphi }-\widehat{P}_{m,n+1}e^{-i\varphi }\big ),\\ -\frac{v_0}{r}\sin (\theta -\varphi )\,\partial _{\theta '} \;&\leadsto \; -\frac{v_0}{2r}\,n\big (\widehat{P}_{m-1,n}e^{+i\varphi }-\widehat{P}_{m+1,n}e^{-i\varphi }\big ). \end{aligned}$$

Observing that $\cos (\theta -\varphi )e^{in\theta '} =\tfrac{1}{2}\bigl (e^{i(n+1)\theta '}+e^{i(n-1)\theta '}\bigr )e^{-i\varphi }$, the cosine term in Equation (12), upon projection, maps as

$$ v_0\cos (\theta '-\varphi )\,\partial _r \delta \tilde{P} \leadsto \frac{v_0}{2}\Big [ e^{+i\varphi }\, \partial _r\!\big (k\,\widehat{P}_{m,n-1}(k,t)\,J_0(kr)\big ) + e^{-i\varphi }\, \partial _r\!\big (k\,\widehat{P}_{m,n+1}(k,t)\,J_0(kr)\big ) \Big ].$$

The radial operator diagonalises such that $\widehat{\mathcal {L}}_r\!\rightarrow \!\lambda _{r}(k)$ with

$$ \lambda _{r}(k)= -k^{2}D_{t} + \rho _{0}k^{2}\widehat{F}(k),\qquad D_{t}:=\frac{\sigma ^{2}}{2},\qquad \widehat{F}(k)=2\pi \int _{0}^{\infty }rF(r)J_{0}(kr)\,dr . $$

Angular diffusion contributes $-D_\theta (m^2+n^2)$. Collecting terms, projection of Equation (12) produces, for each (*m*, *n*),

13 $$\begin{aligned} \partial _t\big (k\widehat{P}_{mn}J_0\big )&= kJ_0\Big (\lambda _r(k)-D_\theta (m^2+n^2)\Big )\widehat{P}_{mn} \end{aligned}$$

14 $$\begin{aligned}&\quad + \frac{v_0}{2r}\,m\Big (k\widehat{P}_{m,n-1}J_0\,e^{+i\varphi }-k\widehat{P}_{m,n+1}J_0\,e^{-i\varphi }\Big )\end{aligned}$$

15 $$\begin{aligned}&\quad - \frac{v_0}{2r}\,n\Big (k\widehat{P}_{m-1,n}J_0\,e^{+i\varphi }-k\widehat{P}_{m+1,n}J_0\,e^{-i\varphi }\Big )\end{aligned}$$

16 $$\begin{aligned}&\quad + \frac{v_0}{2}\Big [ e^{+i\varphi }\,\partial _r\!\big (k\widehat{P}_{m,n-1}J_0\big ) +e^{-i\varphi }\,\partial _r\!\big (k\widehat{P}_{m,n+1}J_0\big ) \Big ]. \end{aligned}$$

To eliminate dependence on $\varphi$, define the next projection

$$\langle f\rangle _\varphi :=\frac{1}{2\pi J_0(kr)}\int _{0}^{2\pi } f(\varphi )\,e^{ik r\cos \varphi }\,d\varphi .$$

Noting that $\langle e^{\pm i\varphi }\rangle _\varphi =\pm i\,J_1(kr)/J_0(kr)$, $\langle 1\rangle _\varphi =1$, and $J_0'(x)=-J_1(x)$. Collecting terms, and using the Ansatz $\widehat{P}_{mn}(k,t)=\widehat{A}_{mn}(k)\,e^{\lambda t}$, for each (*m*, *n*) the evolution is given by

17 $$\begin{aligned} \bigl [\lambda -\lambda _{r}(k)+D_\theta (m^{2}+n^{2})\bigr ]\,\hat{P}_{mn} +\beta \!\bigl ( \hat{P}_{m-1,n}-\hat{P}_{m+1,n} -\hat{P}_{m,n-1}+\hat{P}_{m,n+1} \bigr )=0. \end{aligned}$$

Here, we have used the approximation that $kr \approx 1$ so that $v_0 k J_1(kr)/J_0(kr) \approx v_0 k^2 r/2$. The constant $\beta$ is therefore given by $\beta = k v_0/2$.

#### The Isotropic Block, $(m,n)=(0,0)$

Since $\hat{P}_{-1,0} = \hat{P}_{1,0}^{*}$ and $\hat{P}_{0,-1} = \hat{P}_{0,1}^{*}$, the isotropic mode decouples, so that

$$ \lambda _{0}(k)=\lambda _{r}(k) = -k^{2}D_{t}+\rho _{0}k^{2}\widehat{F}(k). $$

A clustering instability appears when $\lambda _{0}(k)>0$, *i.e.*,

18 $$\begin{aligned} \rho _{0}\widehat{F}(k)\;>\;D_{t}=\frac{\sigma ^{2}}{2}. \end{aligned}$$

In this case, an instability arises through the eigenvalue $\lambda _0(k)$ as it balances translational diffusion against the Fourier transform of the attraction-repulsion kernel. If the condition in Equation (18) occurs, then density modulations at that wavenumber grow exponentially. In that case, particles aggregate into clusters that are sill isotropic in angle. It is only after this clustering sets in that polar patterns can arise due to contributions from $\beta$.

#### The First Anisotropic Instability, $(m,n)=\pm 1,0$

The $4\times 4$ block spanned by $\{(1,0),(-1,0),(0,1),(0,-1)\}$ carries the minimal information needed to distinguish front from back in anisotropic patterns, therefore it will be referred to as the first anisotropic instability. The dominant eigenvalue is given by

$$ \lambda _{\pm 1}(k) = \lambda _{0}(k) - D_\theta + \frac{\beta ^{2}}{\lambda _{0}(k)+D_\theta }, $$

with $\beta =k v_{0}/2$ as before, and $D_\theta =\sigma ^{2}/2$. Positivity of $\lambda _{\pm 1}$ is equivalent to the condition that $\lambda _{0}(k)>D_\theta$ as well as $\beta ^{2}>D_\theta \bigl [\lambda _{0}(k)-D_\theta \bigr ]$. Now, substitution of $\lambda _{0}$ and $\beta$ produces,

19 $$\begin{aligned} \rho _{0}\widehat{F}(k)&> \frac{\sigma ^{2}}{2}\! \Bigl (1+\frac{1}{k^{2}}\Bigr ),\end{aligned}$$

20 $$\begin{aligned} k^{2}v_{0}^{2}&> \frac{\sigma ^{2}}{2}\! \Bigl [2\rho _{0}\widehat{F}(k)-\sigma ^{2} -\frac{\sigma ^{2}}{k^{2}v_{0}^{2}}\Bigr ]. \end{aligned}$$

At this point, we assume a large-Péclet regime: for the most unstable wavenumber $k_{\max }$, $k_{\max }v_{0}\gg \sigma$. The large Péclet limit provides an analytically tractable regime describing persistent active motion, and is commonly used in active matter with persistent movement Tzella and Vanneste (2015). Note that this approximation holds only for the finite-wavenumber regime in which the instability occurs, *i.e.*, where $k v_0 \gg \sigma$ and the neglected term remains asymptotically small. Dropping the $k^{-2}v_{0}^{-2}$ terms simplifies the condition above to

21 $$\begin{aligned} \rho _{0}\widehat{F}(k)>\frac{\sigma ^{2}}{2}, \end{aligned}$$

22 $$\begin{aligned} k^{2}v_{0}^{2}>\sigma ^{2}\bigl [2\rho _{0}\widehat{F}(k)-\sigma ^{2}\bigr ]. \end{aligned}$$

The condition in Equation (21) confirms that dipolar order can arise only after the focal cell’s neighbourhood becomes radially inhomogeneous, whereas the condition in Equation (22) states that ballistic advection must outrun rotational diffusion, i.e. the angular Péclet number, $\textrm{Pe}_{\theta }=kv_{0}/\sigma$, exceeds $\sqrt{2\rho _{0}\widehat{F}(k)-1}$.

### Second-Order Anisotropy: Quadrupole Instability

Front-back structure first appears when $|m|+|n|=1$. To capture growth modes involving lateral patterns, one must allow for quadrupolar variations proportional to $\sin 2\Theta$, so that there are modulations at $\Theta =\pm \pi /2$. In terms of the Fourier indices (*m*, *n*) of Equation (11), $\sin 2\Theta$ corresponds to the pair $(m,n)=(1,-1)$ and $(m,n)=(-1,1)$. This defines the second anisotropic instability, $|m|+|n|=2$. Because the linear convective coupling links each mode to its four nearest neighbours, the smallest closed subset that contains, for instance, $(1,-1)$ is

23 $$\begin{aligned} \mathscr {B}_2=\bigl \{(1,-1),\,(0,-1),\,(1,0)\bigr \}. \end{aligned}$$

#### Linearised System

Writing ${\textbf {g}} =(g_{1,-1},\,g_{0,-1},\,g_{1,0})^{\!\top }$ and defining, $\alpha _1=\lambda -\lambda _r(k)+2D_\theta$, $\alpha _d=\lambda -\lambda _r(k)+D_\theta$, and $\beta =k v_0/2$, Equation (11) restricted to $\mathscr {B}_2$ reads $M_2(\lambda ){\textbf {g}}={\textbf {0}}$ with

24 $$\begin{aligned} M_2(\lambda )= \begin{pmatrix} \alpha _1 & \beta & \beta \\ -\beta & \alpha _d & 0 \\ -\beta & 0 & \alpha _d \end{pmatrix}. \end{aligned}$$

Non-trivial solutions require $\det M_2(\lambda )=0$. Taking the determinant and factorising gives

25 $$\begin{aligned} (\lambda -\lambda _r+D_\theta ) \bigl [(\lambda -\lambda _r)^2 +3D_\theta (\lambda -\lambda _r) +2D_\theta ^{2}+2\beta ^{2}\bigr ] = 0. \end{aligned}$$

The first factor reproduces the dipole growth rate from the previous section, $\lambda _{\textrm{dip}}(k)$, and the quadratic bracket contains the new side-pattern roots.

#### Eigenvalues and Instability Threshold

Solving the quadratic for the side-pattern roots from Equation (25) yields

26 $$\begin{aligned} \lambda _{\textrm{side},\pm }(k) =\lambda _r(k)-\tfrac{3}{2} D_\theta \;\pm \;\tfrac{1}{2}\sqrt{\,D_\theta ^{2}+8\beta ^{2}\,}. \end{aligned}$$

These eigenvalues remain real, and oscillatory growth does not arise in this block. Substituting $\lambda _r(k)$ as before produces the threshold

27 $$\begin{aligned} \rho _0\widehat{F}(k) > \frac{\sigma ^2}{2} + \frac{1}{2k^2}\left( \frac{3\sigma ^2}{2} - \sqrt{\frac{\sigma ^4}{4} + 8\beta ^2}\right) \end{aligned}$$

When this instability arises, the two modes, $(1,-1)$, and $(-1,1)$ combine into $\cos (\theta -\theta ')\sin (\theta -\varphi ) \propto \sin 2\Theta$ after setting $\varphi =\theta +\Theta$. Therefore a positive $\lambda _{\text {side},+}$ produces lobes at $\Theta =\pm \pi /2$ in the neighbour distribution and, via the kernel $f_{\textrm{ar}}(r,\Theta )$, drives a quadrupolar component $\propto \sin 2\Theta$ in the velocity-velocity correlation $C(r,\Theta ,t)$. Finally, if $\lambda _{\pm 1}(k)$ is already positive, the dipole modes feed the quadrupole through the same $\beta$-coupling. That nonlinear reinforcement falls beyond the present linear analysis but is physically plausible.

### A Minimal Dipolar Kernel that Suppresses Dipolar Patterns

A converse question to the one that have been asked so far, is whether observing a kernel that looks largely isotropic must mean that the interaction kernel must be isotropic. The answer to this question is no, and, as will be shown in this section, the reason lies in a strict hierarchy of angular instabilities. Before embarking on this analysis, it is worth noting that clean side-by-side patterns, as instabilities of the linearised system, cannot occur with a radially isotropic interaction force alone. This is because the quadrupolar branch responsible for the lateral lobes in Equation (26) becomes positive only after the density drive has surpassed its $3D_{\theta }/2$ threshold, whereas the dipolar branch requires only a threshold of $D_{\theta }$. Now, to explore how isotropically-looking patterns can emerge from a non-isotropic interaction force, we consider a dipolar (*i.e.* front-back) interaction force, $f_{\textrm{ar}}$. The arguments in this section will show conditions under which such a mechanism can suppress the dipolar instability that would normally be produced by a purely isotropic attraction–repulsion kernel.

#### Kernel with a Single Dipole Harmonic and its Fourier Transform

Consider an interaction kernel that contains only the monopole and a single dipole harmonic,

28 $$\begin{aligned} f_{\!\text {ar}}(r,\phi )=F_0(r)+\varepsilon \,F_1(r)\cos \phi , \qquad 0<|\varepsilon |\ll 1. \end{aligned}$$

The small parameter $\varepsilon$ measures the strength of a front-back bias relative to the isotropic attraction. Consider again the expansion of perturbations of $\tilde{P}$ in a Fourier-Bessel basis (as done in Sections 3.1 and 3.2. As before, the purely radial contribution appears in every block and gives a baseline growth rate $\lambda _r(k)=-k^{2}D_t+\rho _0k^{2}\widehat{F}(k)$. Projecting this common term onto a monopole, $p=0$, *i.e.*, taking the inner product with $\mathrm e^{\mathrm i0\phi }$, yields the familiar leading eigenvalue. This term, being proportional to the zeroth harmonic $F_0$, is then inherited by every angular sector of the linear operator.

Introducing the dipolar kernel in Equation (28) now adds a first angular harmonic but no higher ones. Writing $\cos \phi =\frac{1}{2}(\mathrm e^{\mathrm i\phi }+\mathrm e^{-\mathrm i\phi })$ shows that the only new Fourier coefficients are $F_{\pm 1}=\varepsilon F_1/2$. When the interaction matrix is projected onto $p=\pm 1$, *i.e.*, onto the dipoles, this additional coefficient contributes a diagonal shift

$$ \alpha (k)=\frac{\rho _0k^{2}\varepsilon }{2}\,\widehat{F}_1(k), $$

where, analogously to the sections before, $\widehat{F}_1(k)\equiv 2\pi \!\int _0^\infty rF_1(r)J_1(kr)\,dr$. If $\varepsilon \widehat{F}_1 < 0$, then $\alpha (k)<0$. In this case the dipole growth rate is depressed. *Vice versa*, a positive value reinforces it.

#### Updated Dipole and Quadrupole Eigenvalue Blocks

Since for each radial wave-number *k*, the isotropic sector is unchanged, but the dipole block carries an extra diagonal entry $+\alpha (k)$, it follows that the eigenvalue corresponding to the dipole block is now defined by

29 $$\begin{aligned} \; \lambda _{\text {dip}}(k)=\lambda _r^{(0)}(k)+\alpha (k)-D_\theta \;+\; \frac{\beta ^{2}}{\lambda _r^{(0)}(k)+D_\theta }.\; \end{aligned}$$

Recall that the quadrupole (side) matrix, $M_2(\lambda )$, of Section 3.2 does not involve the $p=\pm 1$ modes, so its eigenvalues are unaffected and are given by Equation (26).

#### Instability Conditions for a Suppressed Dipolar Pattern

To find instabilities such that an emergent dipole pattern is suppressed, one must seek wavenumbers for which $\lambda _{\text {side},+}(k)>0$ and $\lambda _{\text {dip}}(k)<0$. Using Equations (26) and (29), it is sufficient that

30 $$\begin{aligned} D_\theta< \lambda _r^{(0)}(k) < D_\theta -\alpha (k). \end{aligned}$$

Equation (30) can hold only if $\alpha (k)<0$, i.e. $\varepsilon \widehat{F}_1(k)<0$. Hence a moderately repulsive dipole component ($\varepsilon F_1<0$) depresses the dipole growth rate below zero while the isotropic drive remains large enough to trigger the quadrupole. Translated to microscopic parameters, the coexistence band for a suppressed dipole pattern instability to arise is defined by

31 $$\begin{aligned} \varepsilon \widehat{F}_1(k) < -2\widehat{F}_0(k) + \frac{\sigma ^2}{\rho _0}\left( 1 + \frac{1}{k^2}\right) . \end{aligned}$$

Increasing $|\varepsilon |$, *i.e.* strengthening the dipolar repulsion, widens the parameter window in which dipole pattern instabilities can be suppressed. Put together, anisotropy in the interaction kernel is required, but its sign and magnitude decide whether the observable correlation field ends up anisotropic, and also which multipole wins. A dipolar kernel can, counter-intuitively, produce a quadrupolar correlation pattern that is not hard-wired in the force itself.

### Numerical Validation

To test the instability predictions of Sections 3.1-3.3 on empirical trajectories, a classic framework with an attraction-repulsion kernel (D’Orsogna et al. 2006) is used as a specific example of an SPP model in the form of Equation (2). We compare the resulting stationary correlation PDE, and direct simulations with the resulting correlation fields. The model is given by

32 $$\begin{aligned} S({\textbf {v}}_i)=\alpha -\beta |{\textbf {v}}_i|, \qquad f_{\textrm{ar}}(r)= \mathcal {M}(r), \qquad \mathcal {M}(r)= \frac{C_R}{L_R}e^{-r/L_R}-\frac{C_A}{L_A}e^{-r/L_A}, \end{aligned}$$

with $\alpha =\beta$ so that the preferred speed is unity. Crucially, the attraction-repulsion force is strictly radial. We simulate *N* agents in a periodic square of side $L=70$ using an Euler-Maruyama scheme with $\Delta t=0.02$ for 2000 time steps, using the first 500 time steps as burn-in. The number of agents is chosen to achieve the target density $\rho _0=N/L^2$ in each regime. Empirical correlations are measured in each focal particle’s frame:

$$ C_{\textrm{emp}}(r,\theta )= \big \langle \,\hat{{\textbf {v}}}_i\!\cdot \!\hat{{\textbf {v}}}_j\,\big \rangle _{\,j:\, r_{ij}\in [r,r+\Delta r],\ \theta _{ij}\in [\theta ,\theta +\Delta \theta ]}, $$

where $\theta _{ij}$ is the polar angle of ${\textbf {r}}_{ij}$ relative to $\hat{{\textbf {v}}}_i$ and neighbours are binned on a polar grid $(r,\theta )$. For the isotropic part of the force we use a Morse form $F_0(r)=\mathcal {M}(r)$, and for the anisotropic dipole we choose $F_1(r,\phi )$ as before, so that

$$ \widehat{F}_0(k)=2\pi \!\left[ \frac{C_R/L_R^2}{(L_R^{-2}+k^2)^{3/2}} -\frac{C_A/L_A^2}{(L_A^{-2}+k^2)^{3/2}}\right] ,\qquad \widehat{F}_1(k)=2\pi A_1\,\frac{k}{(L_1^{-2}+k^2)^{3/2}}. $$

This is used to compute the dispersion curves for the $\ell =0,1,2$ branches and identify the most-unstable wavenumber $k_*$ used to select the branch for each regime. For the PDE comparison we solve, in angular Fourier components, the steady radial boundary-value problems implied by Equation (7). Each mode $C_m(r)$ satisfies a second-order ODE on $r\in [0,R]$ with $R=L/2$, driven by a spatially constant source, $S_m(r)$. Boundary conditions are regularity at $r=0$ (Neumann for $m=0$, $C_m(0)=0$ for $m\ge 1$) and $C_m(R)=0$. We discretise with a uniform radial grid using second-order centered differences and solve the resulting banded linear systems directly. For the quadrupolar column we superpose the $m=0$ and $m=2$ solutions with the mixing ratio $u_2/u_0$ evaluated at $k_*$ from the side branch eigenvector; other columns display a single mode ($m=0$ or $m=1$). Fields are normalized to unit maximum absolute value before plotting in the co-moving frame.

**Fig. 1:**
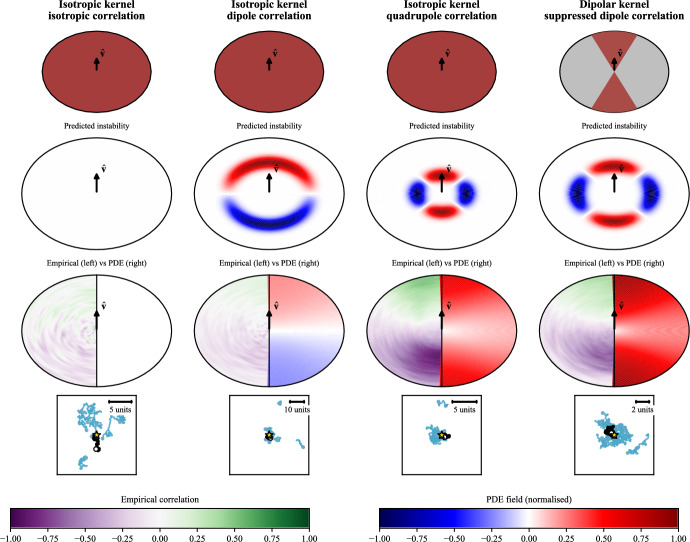
Numerical validation of the instability theory. Top row: schematics of the interaction kernels. Second row: for each regime the most unstable harmonic predicted by the dispersion analysis as $C(r,\theta )$ on a ring. Third row: left semicircles show the empirical correlation field from simulations and right semicircles show the stationary solution of the linear PDE evaluated with the same parameters; the arrow indicates the focal heading $\hat{{\textbf {v}}}$. Bottom: representative focal and neighbor trajectories with scale bars. Simulation parameters are listed in Table 1. Note that the PDE predictions align well with the empirical dipole pattern in the second column, but fail to capture the exact structure in the quadrupole and suppression regimes, highlighting that leading-order linear instabilities are not always a reliable proxy for the fully developed PDE solutions

We set $L_R=0.5$, $L_A=2.0$, $C_R=1.0$, $C_A=0.95$, and $v_0=1$. Further, the parameter choices listed in Table 1 are designed to isolate the behaviours discussed in Sections 3.1-3.3. Figure 1 shows the computed correlations and empirical trajectories for each of these parameter regimes. In the isotropic kernel at low density and high noise, the correlation field is nearly flat, consistent with no unstable modes. As density increases, the dipole branch crosses zero, producing a faint but detectable front-back asymmetry. At still higher density the quadrupolar branch dominates, leading to side lobes visible in the correlation field. Finally, when a weak anisotropic dipolar term is added to the kernel, the dipole instability is suppressed: the empirical correlation is reduced in amplitude, consistent with the predicted suppression window.

In Figure 1, the agreement between the predicted linear mode of the PDE and empirical correlation is clearest in the isotropic-dipole case, where the PDE prediction reproduces the front-back bias seen empirically. In contrast, for the isotropic-quadrupole and dipole-suppression regimes the linearised PDE fields do not resemble the full empirical correlations. This mismatch is expected as the PDE fields are constructed from the leading linear instability alone, whereas the empirical correlations reflect nonlinear saturation, harmonic mixing, and finite-density effects. Linear theory is therefore a good proxy only when a single branch is near threshold, but not when multiple instabilities interact or are strongly subcritical. Nevertheless, the qualitative features of growing magnitude of the growth coefficient and the subsequent suppression are recapitulated by the linear theory in Figure 1.

**Table 1:** Parameter values used for the simulation of interacting agents according to the model in Equation (32)

Case	Kernel	$\rho _0$	$\sigma$	Extra parameters
Isotropic / isotropic correlation	$F_0$	0.08	0.90	–
Isotropic / dipolar correlation	$F_0$	0.35	1.0	–
Isotropic / quadrupolar correlation	$F_0$	0.90	0.45	–
Dipolar kernel / suppressed dipole	$F_0+F_1$	0.55	0.80	$\varepsilon =-0.50,\;A_1=1.0,\;L_1=1.0$

To verify the extent to which the linearly predicted unstable modes are present in the empirical correlation fields that are computed for each of the regimes, the empirical correlation fields from Figure 1 are decomposed orthogonally into angular harmonics, such that

$$\begin{aligned} C(r,\theta )=a_0(r)+\sum _{m\ge 1}a_m(r)\cos (m\theta ). \end{aligned}$$

**Fig. 2:**
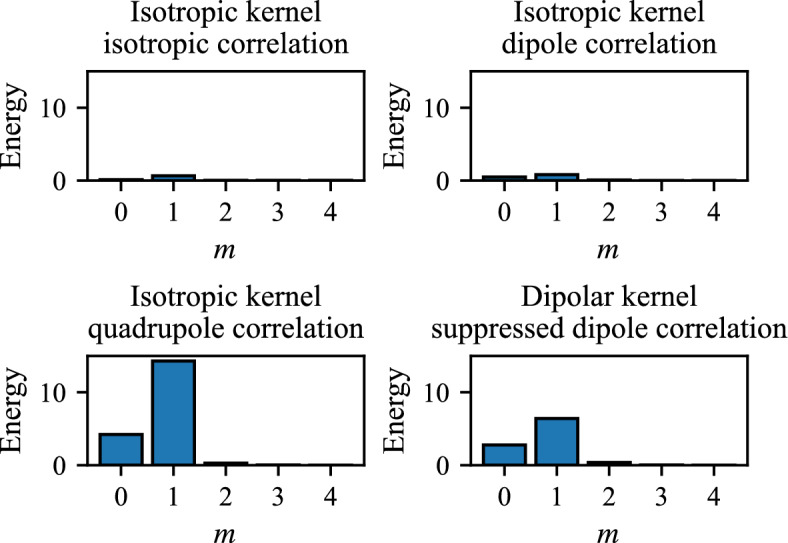
Angular-harmonic decomposition of empirical correlations. For each regime, the empirical correlation field from Fig. 1 is decomposed orthogonally into angular harmonics

The bar plots in Figure 2 show the contributions, $E_m$, of each harmonic, which are defined as

33 $$\begin{aligned} E_0=2\pi \int a_0(r)^2\,r\,dr,\qquad E_m=\pi \int a_m(r)^2\,r\,dr\ (m\ge 1). \end{aligned}$$

Identifying the contributions of each harmonic isolates which harmonic is present in each regime. The bar plots of Figure 2 confirm the pattern shown by the sequence of instabilities discussed in Figure 1. In the regime predicted to host a dipole instability, the $m=1$ contribution remains subdominant, consistent with the linear growth rate being weak at the chosen parameters. In the quadrupole regime, higher modes contribute alongside $m=2$, showing that the empirical correlation saturates as a mixture rather than a pure eigenmode. The dipole-suppression case shows that the dominant eigenmodes are suppressed relative to the quadrupolar correlation regime.

## Effect of Velocity Alignment on Emergent Correlations

Sections 2 and 3 showed that isotropic attraction-repulsion kernels alone can generate emergent anisotropic correlations. While this isolates the role of pure attraction and repulsion anisotropy, many examples of interacting agents in biology include velocity alignment explicitly in response to neighbours (Michael Te and Wittkowski 2025). To explore how such an extra model term influences the emergence of correlations on top of attraction-repulsion dynamics, here we incorporate velocity alignment in the simplest, linear, form compatible with the model used above. We extend the single-particle dynamics in Equation (1) by adding an additional force, $F_{\textrm{align}}$ that rotates the heading of cell *i* toward the heading of *j*,

34 $$\begin{aligned} F_{\textrm{align}} = \frac{v_0}{N}\sum _{j\ne i} f_{al}(r_{ij},\varphi _{ij})\,(I-\hat{u}_i\hat{u}_i^\top )\hat{u}_j, \end{aligned}$$

where $\hat{u}_i=v_i/|v_i|$. The projector, $(I-\hat{u}_i\hat{u}_i^\top )$, removes the component along $\hat{u}_i$, so alignment only rotates headings and preserves speed. For simplicity, we parameterise the distance- and angle-dependent alignment density, $f_{\text {al}}$, by assuming that it can be written as

35 $$\begin{aligned} f_{al}(r,\theta )=\eta _0 F_0(r)+\eta _1\,\varepsilon F_1(r)\cos \theta , \end{aligned}$$

*i.e.* so that the Bessel transforms computed for the alignment kernel will be proportional to those of the attraction-repulsion kernel, $f_{\text {ar}}$. The alignment drift in Equation (34) will now contribute to the Fokker-Planck equation,

36 $$\begin{aligned} \partial _t C \;=\; -v_0\,\partial _r C + D_r\!\left( \partial _r^2 + r^{-1}\partial _r\right) C + \frac{D_\theta }{r^2}\partial _\theta ^2 C - 2\gamma \,C \;+\; f_{ar} + f_{al}, \end{aligned}$$

see Appendix E for details. Thus, alignment enters additively, with the same harmonic content as $f_{ar}$, and the Fourier-Bessel block structure of Section 3 is unchanged. Alignment changes only by having the first harmonic in $f_{al}$ contributing a diagonal shift to the dipole block, exactly as a dipolar kernel, $f_{\text {ar}}$ does in Equation (28), while leaving the side matrix of Section 3.2 unaltered at leading order. Defining $\widehat{A}_1(k)$ as

$$ \widehat{A}_1(k)=2\pi \int _0^\infty r\,\big ( F_1(r)\big )J_0(kr)\,dr, $$

the dipole eigenvalue becomes

37 $$\begin{aligned} \lambda _{\textrm{dip}}(k) \;=\; \lambda _r^{(0)}(k) \;+\; \alpha _{ar,1}(k)\;+\; \alpha _{al,1}(k) \;-\; D_\theta \;+\; \frac{\beta ^2}{\lambda _r^{(0)}(k)+D_\theta }, \end{aligned}$$

where

38 $$\begin{aligned} \alpha _{al,1}(k)=\frac{\rho _0 k^2}{2}\eta _1 \varepsilon \,\widehat{A}_1(k), \qquad \alpha _{ar,1}(k)=\frac{\rho _0 k^2}{2}\,\varepsilon \,\widehat{F}_1(k), \end{aligned}$$

and $\beta =kv_0/2$ as before. The side branch eigenvalues remain the same, as no new terms enter. Two consequences follow immediately. First, the thresholds needed to induce a dipole instability shift, as any $\eta _1\ne 0$ simply replaces the dipole shift that occurs due to attraction-repulsion by $\alpha _{ar,1}\!+\alpha _{al,1}$ in Equation (37). A positive alignment term given by $\eta _1$ therefore raises $\lambda _{\textrm{dip}}$ across 0 at lower $v_0$ or density, whereas a negative $\eta _1$ (*i.e.*, anti-alignment) depresses it. Second, the parameter window in which dipolar instabilities are depressed can be either created or erased. Because the side matrix, $M_2$, is unchanged, the inequality that guarantees this window is still

39 $$\begin{aligned} D_\theta \;<\; \lambda _r^{(0)}(k) \;<\; D_\theta - \big (\alpha _{ar,1}(k)+\alpha _{al,1}(k)\big ), \end{aligned}$$

*cf.* Equation (30) with $\alpha \mapsto \alpha _{ar,1}+\alpha _{al,1}$. Thus, anti-alignment in the front-back sector ($\eta _1<0$) widens this window even when $f_{ar}$ is isotropic, while pro-alignment narrows or destroys it. In this minimal extension, alignment can selectively modulate the dipole instability with no change to the side matrix, and the final resulting pattern hinges on the sign and magnitude of $\alpha _{al,1}(k)$.

**Fig. 3:**
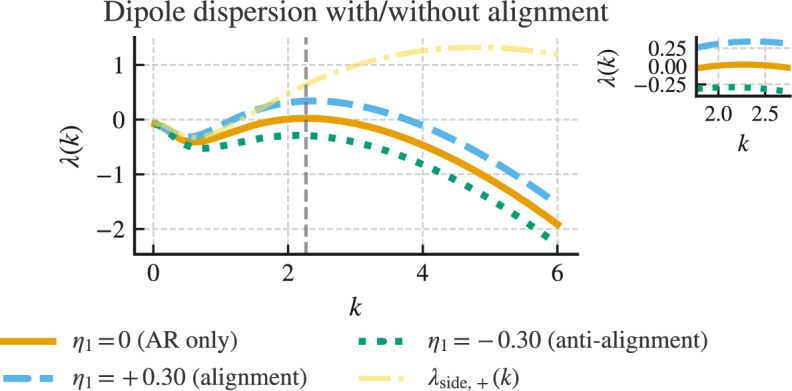
Dispersion curves, $\lambda (k)$, for the dipole branch with no alignment $(\eta _1=0)$, with alignment $(\eta _1>0)$ and anti-alignment $(\eta _1<0)$, together with the side branch $\lambda _{\text {side},+}(k)$. Alignment shifts only the dipole curve by $\alpha _{\textrm{al},1}(k)$ in Eq. (37), while $\lambda _{\text {side},+}(k)$ is unaffected at leading order. Inset: zoom near $k^*$

As an illustration, Figure 3 shows the dispersion relation for the same parameters as the suppressed dipole regime in Figure 1 with a weak dipolar component in the attraction-repulsion kernel ($\epsilon = -0.5$, $A_1 = 1.0$, $L_1 = 1.0$). At these parameters, the Morse attraction–repulsion kernel alone produced an unstable quadrupole instability as well as an unstable dipole instability (with a smaller growth rate). We now introduce a separate dipolar alignment term, with amplitude $\eta _1$. As discussed previously, this term can selectively shift the dipole eigenvalue without affecting the quadrupole growth rate. We therefore explore $\eta _1 \in \{0,\, +0.3,\, -0.3\}$, corresponding respectively to: no alignment, alignment, and anti-alignment, while all other parameters remain fixed. For $\eta _1=0$, the dispersion relation $\lambda _{\textrm{dip}}(k)$ exhibits a maximum of zero near $k\simeq 2.1$, indicating a critical point for an emergent dipole instability, as before. For $\eta _1>0$ (alignment), $\alpha _{\textrm{al},1}(k)>0$ and the dipole curve shifts upward, slightly enhancing this mode. For $\eta _1<0$ (anti-alignment), $\alpha _{\textrm{al},1}(k)<0$ and the dipole curve shifts downward while the side branch $\lambda _{\textrm{side},+}(k)$ remains unchanged, thereby creating a distinct band of *k* for which

$$ \lambda _{\textrm{dip}}(k)< 0 < \lambda _{\textrm{side},+}(k), $$

corresponding to a parameter regime in which the dipole instability is even further suppressed. Put together, this demonstrates that an alignment term can selectively dampen or strengthen the dipole instability while leaving higher modes largely unchanged, thus fundamentally changing the linear stability properties of the emergent correlation field.

## Identifiability of Interaction Anisotropy from Correlation Fields

A natural question arising from the preceding analysis concerns identifiability. In its most general meaning, an observable is said to identify a parameter if the mapping from the model parameters to the observable is injective Simpson and Baker (2026). In the present setting, the relevant observable is the stationary two point velocity correlation field, $C(r,\theta )$, governed by Equation (7). An important question is therefore whether anisotropy in the interaction kernel $f_{ar}(r,\theta )$ can be uniquely recovered from *C*. To illustrate why this is not the case in general, we consider a worked example where we consider the angular modes restricted to a given radius, $r=r_*$. We begin from the steady form of Equation (7),

40 $$\begin{aligned} 0 = -v_0 \partial _r C^*+ D_r\left( \partial _r^2+r^{-1}\partial _r\right) C^*+ \frac{D_\theta }{r^2}\partial _\theta ^2 C^*- 2\gamma C^*+ \rho \,g(r,\theta )f_{ar}(r,\theta ), \end{aligned}$$

and for the sake of argument consider the simplest homogeneous setting in which the neighbour distribution is isotropic, *i.e.*, $g(r,\theta )=g_0(r)$, and furthermore restrict the interaction kernel to the form $f_{ar}(r,\theta )=F_0(r)+\varepsilon F_1(r)\cos \theta .$ If the radial variation of $C^*$ across this annulus is small compared with its angular variation, at $r=r_*$, Equation (7) at leading order reduces to

41 $$\begin{aligned} \frac{D_\theta }{r_*^2}\partial _\theta ^2 C^*(r_*,\theta ) - 2\gamma C^*(r_*,\theta ) + \rho \,g_0(r_*)\Bigl (F_0(r_*)+\varepsilon F_1(r_*)\cos \theta \Bigr ) = 0. \end{aligned}$$

Expanding the stationary correlation on this shell in $\theta$ and matching the zeroth and first angular harmonics gives

42 $$\begin{aligned} -2\gamma \,C_0(r_*)+\rho \,g_0(r_*)F_0(r_*)=0, \end{aligned}$$

and

43 $$\begin{aligned} -\left( 2\gamma +\frac{D_\theta }{r_*^2}\right) C_1(r_*) + \rho \,g_0(r_*)\varepsilon F_1(r_*)=0. \end{aligned}$$

Hence,

44 $$\begin{aligned} C_0(r_*)=\frac{\rho \,g_0(r_*)F_0(r_*)}{2\gamma }, \qquad C_1(r_*)= \frac{\rho \,g_0(r_*)\,\varepsilon F_1(r_*)}{2\gamma +D_\theta /r_*^2}. \end{aligned}$$

We conclude that, even in this simplified homogeneous setting, a measured value of $C_1(r_*)$ does not determine the anisotropy amplitude $\varepsilon$ uniquely, but only this reduced parameter combination. In particular, variations in the dipolar radial profile $F_1(r_*)$ or in the effective angular damping $2\gamma + D_\theta /r_*^2$ can compensate for changes in $\varepsilon$ and produce the same observed dipole amplitude. Although this example does not constitute a formal identifiability analysis, it provides a simple heuristic illustration of how natural parameter degeneracies can arise when attempting to infer interaction anisotropy from angular harmonics of the correlation field. It is important to note that the simplified homogeneous setting considered here removes the spatial coupling mechanisms analysed in previous sections, which introduce further coupling between modes.

## Discussion

By deriving a kinetic theory-based closed-form PDE from a canonical SPP model, this work establishes conditions under which intrinsic directional biases can be reliably distinguished from emerging anisotropy in the velocity correlations between two interacting particles. A linear stability analysis revealed that front-back or lateral biases may arise spontaneously and various kinetic mechanisms can contribute to the emergence or suppression of such instabilities. This work therefore complements recent developments in modelling of active matter (Jiang et al. 2017; Brunton et al. 2016; Messenger et al. 2022) providing an explicit analytical framework for the angular structure of correlations. These findings underscore the importance of ruling out alternative explanations before concluding that directional forces drive observed anisotropic behaviors. In fact, the theory developed in this paper can be used as the basis for statistical testing whether observed velocity correlations in collectively migrating agents are due to an inherent anisotropy in the interaction kernel. This is because, the spectrum of the operator that dictates the evolution of the correlation field specifies which harmonics are expected to be stable under isotropic interactions. Angular decomposition of observed correlations would identify which modes are present. Using a noise model, the magnitude of such modes can be tested against the null model of what can be expected given an isotropic kernel. Developing such tests is an obvious next step for applications to experimental data.

We emphasise that, although the analysis was presented using a canonical self-propelled particle formulation, the mechanism through which isotropic interactions forces lead to anisotropic observed correlations in this work is not specific to the particular interaction kernel used in the simulations. The attraction–repulsion force $f_{ar}(r,\theta )$ is a general interaction term which can capture a wide class of pairwise interaction laws as long as the dynamics are not strongly damped. For example, Lennard–Jones-type interactions can be written as a purely radial force, *F*(*r*) and therefore fall directly within the isotropic kernel analysed in this work. Similarly, many commonly used models of collective motion can be expressed in the same structural form, including attraction–repulsion particle models, Vicsek-type alignment models (when written in continuous-time form), and active Brownian particle systems with short-range interactions. This work made several structural assumptions that define the range of systems to which the analytical applies directly. First, interactions are assumed to be pairwise additive with a mean-field closure, so models with strong many-body effects or explicit network interactions would require a higher-order kinetic description or closure. Second, the projection onto a constant-speed manifold assumes self-propulsion dynamics that stabilise particle speed; systems with strong inertia or without such speed regulation would therefore require retaining speed fluctuations explicitly. This is possible within the present framework, but requires incorporation of these effects into the governing equations for the velocity and correlation fields. Finally, the linear stability analysis is performed around a spatially homogeneous state, so systems dominated by confinement, external fields, or other imposed spatial structure would require modifications to the correlation equation.

This work can be extended in several ways. First, a formal analysis of nonlinear feedback mechanisms among kinetic processes may elucidate more complex collective behaviors beyond linear stability predictions, and allow for better identification of kinetic phenomena beyond mean field closure. From the perspective of inverse inference, additional information could in principle be obtained from higher-order statistics. For example, three-body correlations, conditional neighbour distributions, or multi-time trajectory statistics may capture geometric structures that are not governed by pair correlations alone. Such observables could potentially help distinguish intrinsic anisotropic interaction kernels from anisotropic correlations that emerge from isotropic forces. Developing inference frameworks that combine the present correlation-based approach with higher-order statistics would therefore be an interesting direction for future work. Second, integrating this kinetic-theory approach into a fully Bayesian inference framework would enable rigorous quantification of uncertainty and facilitate inference of the true underlying interaction kernels. From a hypothesis testing point of view, the angular spectrum of the empirical correlation field could be used to construct hypothesis tests for anisotropy. For example, one could compare the measured amplitudes of the angular harmonics with those expected under a null model of isotropic interactions, estimated using bootstrap or surrogate-data procedures that account for finite sampling and measurement noise. Such tests would allow anisotropic interaction kernels to be distinguished from anisotropic correlations generated by isotropic dynamics. Finally, employing additional biological data, such as tracking cell-cell contacts, cell morphology, or organelle dynamics through fluorescent microscopy, could provide deeper mechanistic insights and suggest plausible biological mechanisms giving rise to the observed interaction laws.

As a final comment, the multipole-instability mechanism discussed in this paper is directly analogous to the classical Turing instability in reaction-diffusion systems. There, an isotropic, slowly diffusing ‘activator’ species and a more rapidly diffusing ‘inhibitor’ species select a finite band of spatial wavenumbers. This interaction produces stationary stripes or spots, despite both species and their diffusion being isotropic (Am and Turing 1952). In the Fokker-Planck equation framework, different angular harmonics of an isotropic interaction kernel play the role of distinct ‘species’. The monopole (isotropic drive) supplies the baseline growth of correlations, while the dipole harmonic functions as an angular ‘inhibitor’, selectively damping front-back modes in a wavenumber-dependent fashion. When this dipolar sector is sufficiently suppressed, the quadrupole mode becomes unstable over a finite band of $k$, yielding clear side-aligned correlation ‘lanes’. Hence, anisotropic patterns in the two-point velocity correlation arise not from explicit breaking of rotational symmetry in the forces, but from the interplay of isotropic interaction modes and angular mixing.

## Appendix A: From the *N*-Body Fokker-Planck Equation to a Pair Equation

We begin with the microscopic equations of motion for *N* interacting self-propelled particles:

A1 $$\begin{aligned} \dot{{\textbf {x}}}_i&= {\textbf {v}}_i, \end{aligned}$$

A2 $$\begin{aligned} \dot{{\textbf {v}}}_i&= S({\textbf {v}}_i){\textbf {v}}_i + \frac{1}{N} \sum _{j \ne i} f_{\text {ar}}(r_{ij}, \phi _{ij}) \hat{{\textbf {r}}}_{ij} + \sigma \boldsymbol{\xi }_i(t), \end{aligned}$$

where $\hat{{\textbf {r}}}_{ij} = \frac{{\textbf {x}}_j - {\textbf {x}}_i}{\Vert {{\textbf {x}}_j - {\textbf {x}}_i} \Vert }$ and $\phi _{ij}$ is the angle between ${\textbf {v}}_i$ and ${\textbf {x}}_j - {\textbf {x}}_i$. This stochastic system corresponds to the *N*-particle distribution function $f_N({\textbf {x}}_1, {\textbf {v}}_1, \dots , {\textbf {x}}_N, {\textbf {v}}_N, t)$. According to the Itô-Kolmogorov forward equation, this density evolves under the *N*-body Fokker-Planck equation:

A3 $$\begin{aligned} \frac{\partial f_N}{\partial t}&+ \sum _{i=1}^N \nabla _{{\textbf {x}}_i} \cdot ({\textbf {v}}_i f_N) + \sum _{i=1}^N \nabla _{{\textbf {v}}_i} \cdot \left[ \left( S({\textbf {v}}_i){\textbf {v}}_i + \frac{1}{N} \sum _{j \ne i} f_{\text {ar}}(r_{ij}, \phi _{ij}) \hat{{\textbf {r}}}_{ij} \right) f_N \right] \nonumber \\&= \frac{\sigma ^2}{2} \sum _{i=1}^N \Delta _{{\textbf {v}}_i} f_N. \end{aligned}$$

### Reduced Distributions

We define the one-particle and two-particle reduced distribution functions as marginals of $f_N$:

A4 $$\begin{aligned} f_1({\textbf {x}}_1, {\textbf {v}}_1, t)&= \int f_N \, d{\textbf {x}}_2 d{\textbf {v}}_2 \dots d{\textbf {x}}_N d{\textbf {v}}_N, \end{aligned}$$

A5 $$\begin{aligned} f_2({\textbf {x}}_1, {\textbf {v}}_1, {\textbf {x}}_2, {\textbf {v}}_2, t)&= \int f_N \, d{\textbf {x}}_3 d{\textbf {v}}_3 \dots d{\textbf {x}}_N d{\textbf {v}}_N. \end{aligned}$$

Our goal is to derive an evolution equation for $f_2$ by integrating the *N*-body equation over particles 3 to *N*.

### BBGKY Equation for $f_2$

Integrating the *N*-body equation over particles $3, \dots , N$ yields:

A6 $$\begin{aligned} \frac{\partial f_2}{\partial t}&+ \nabla _{{\textbf {x}}_1} \cdot ({\textbf {v}}_1 f_2) + \nabla _{{\textbf {x}}_2} \cdot ({\textbf {v}}_2 f_2) \nonumber \\&+ \nabla _{{\textbf {v}}_1} \cdot \left[ S({\textbf {v}}_1){\textbf {v}}_1 f_2 + \frac{1}{N} f_{\text {ar}}(r_{12}, \phi _{12}) \hat{{\textbf {r}}}_{12} f_2 + \frac{1}{N} \sum _{k \ge 3} \int f_{\text {ar}}(r_{1k}, \phi _{1k}) \hat{{\textbf {r}}}_{1k} f_3 \, d{\textbf {x}}_3 d{\textbf {v}}_3 \right] \nonumber \\&+ \nabla _{{\textbf {v}}_2} \cdot \left[ S({\textbf {v}}_2){\textbf {v}}_2 f_2 + \frac{1}{N} f_{\text {ar}}(r_{21}, \phi _{21}) \hat{{\textbf {r}}}_{21} f_2 + \frac{1}{N} \sum _{k \ge 3} \int f_{\text {ar}}(r_{2k}, \phi _{2k}) \hat{{\textbf {r}}}_{2k} f_3 \, d{\textbf {x}}_3 d{\textbf {v}}_3 \right] \nonumber \\&= \frac{\sigma ^2}{2} \left( \Delta _{{\textbf {v}}_1} + \Delta _{{\textbf {v}}_2} \right) f_2. \end{aligned}$$

### Mean-Field (Molecular Chaos) Closure

We now close the hierarchy by approximating the three-particle distribution function using a product of lower-order distributions:

A7 $$\begin{aligned} f_3({\textbf {x}}_1, {\textbf {v}}_1, {\textbf {x}}_2, {\textbf {v}}_2, {\textbf {x}}_3, {\textbf {v}}_3) \approx f_2({\textbf {x}}_1, {\textbf {v}}_1, {\textbf {x}}_2, {\textbf {v}}_2) f_1({\textbf {x}}_3, {\textbf {v}}_3). \end{aligned}$$

Using this, we obtain an approximate expression for the additional interaction term:

A8 $$\begin{aligned} \frac{1}{N} \sum _{k \ge 3} \int f_{\text {ar}}(r_{1k}, \phi _{1k}) \hat{{\textbf {r}}}_{1k} f_3 \, d{\textbf {x}}_3 d{\textbf {v}}_3 \approx \frac{N-2}{N} f_2 \int f_{\text {ar}}(r_{13}, \phi _{13}) \hat{{\textbf {r}}}_{13} f_1({\textbf {x}}_3, {\textbf {v}}_3) \, d{\textbf {x}}_3 d{\textbf {v}}_3. \end{aligned}$$

For large *N*, $\frac{N-2}{N} \approx 1$, so we simplify this further.

### Pair-Density Representation

Define the pair-probability density in terms of relative coordinates:

A9 $$\begin{aligned} P({\textbf {r}}, {\textbf {v}}, {\textbf {v}}', t) = \left\langle \sum _{i \ne j} \delta ({\textbf {r}} - {\textbf {x}}_j + {\textbf {x}}_i) \delta ({\textbf {v}} - {\textbf {v}}_i) \delta ({\textbf {v}}' - {\textbf {v}}_j) \right\rangle . \end{aligned}$$

This is a symmetrized version of $f_2$ written in relative coordinates ${\textbf {r}} = {\textbf {x}}_j - {\textbf {x}}_i$ and velocities ${\textbf {v}}, {\textbf {v}}'$.

### Fokker-Planck Equation

We now obtain the evolution equation for *P* by transforming the equation for $f_2$:

A10 $$\begin{aligned} \partial _t P + ({\textbf {v}}' - {\textbf {v}}) \cdot \nabla _{{\textbf {r}}} P + \mathcal {L}_{{\textbf {v}}}[P] + \mathcal {L}_{{\textbf {v}}'}[P] = \frac{\sigma ^2}{2} \left( \Delta _{{\textbf {v}}} + \Delta _{{\textbf {v}}'} \right) P, \end{aligned}$$

where the operator $\mathcal {L}_{{\textbf {v}}}[P]$ accounts for self-propulsion and interaction drift:

A11 $$\begin{aligned} \mathcal {L}_{{\textbf {v}}}[P] = \nabla _{{\textbf {v}}} \cdot \left( -S({\textbf {v}}){\textbf {v}} - \int f_{\text {ar}}(r, \phi ({\textbf {v}}, {\textbf {r}})) \hat{{\textbf {r}}} \, P({\textbf {r}}, {\textbf {v}}, {\textbf {v}}', t) \, d{\textbf {r}} d{\textbf {v}}' \right) . \end{aligned}$$

This completes the BBGKY reduction to a Fokker-Planck equation for the pair-probability density.

## Appendix B: Conditional Correlation Field $C(r,\theta ,t)$

The objective of this section is to extract a closed evolution equation for the correlation field defined in polar variables centred on a focal particle.

### Geometry of the Co-Moving Frame

For a chosen focal particle *i* align the local *x*-axis with ${\textbf {v}}_i/v_0=\hat{{\textbf {u}}}=(1,0)$. For its neighbour *j* set

A12 $$\begin{aligned} r:=r_{ij}=\Vert {\textbf {x}}_j-{\textbf {x}}_i\Vert ,\qquad \theta :=\angle (\hat{{\textbf {u}}},{\textbf {x}}_j-{\textbf {x}}_i). \end{aligned}$$

Write the headings as $\hat{{\textbf {u}}}=(1,0)$ and $\hat{{\textbf {u}}}'=(\cos \psi ,\sin \psi )$ with $\psi =\varphi -\varphi '$. In this frame ${\textbf {v}}=v_0\hat{{\textbf {u}}}$ and ${\textbf {v}}'=v_0\hat{{\textbf {u}}}'$.

### Pair Density, Distribution Function and Correlation Field

Define the following quantities,

A13 $$\begin{aligned} n(r,\theta ,t)&:= \iint P\,d\varphi \,d\varphi ',\end{aligned}$$

A14 $$\begin{aligned} g(r,\theta ,t)&:= n/\rho ,\end{aligned}$$

A15 $$\begin{aligned} C(r,\theta ,t)&:= \frac{1}{n}\iint (\hat{{\textbf {u}}}\!\cdot \!\hat{{\textbf {u}}}')P\,d\varphi \,d\varphi '. \end{aligned}$$

Here $\rho$ is the bulk (spatially averaged) particle number density, *i.e.*,

$$ \rho \;=\; \int f_1({\textbf {x}},{\textbf {v}},t)\,d{\textbf {v}}. $$

Dividing the pair density $n(r,\theta ,t)$ by this constant scale makes the pair-distribution function $g=n/\rho$ dimensionless. Because $\hat{{\textbf {u}}}\!\cdot \!\hat{{\textbf {u}}}'=\cos \psi \in [-1,1]$ it immediately follows that $-1\le C\le 1$.

### Step 1: Angular Integral Identity

Using $\partial _\varphi ^2\cos \psi =-\cos \psi$ one finds

A16 $$\begin{aligned} \int _0^{2\pi }\!\int _0^{2\pi }\cos \psi \,P\,d\varphi \,d\varphi '=nC. \end{aligned}$$

This identity will be invoked in later steps.

### Step 2: Multiplying the Fokker-Planck Equation by $\cos \psi$

Insert $\cos \psi$ into Equation (A10), integrate over $\varphi ,\varphi '$ and treat each term separately. We catalogue them as (i)-(v) for future reference.

**(i) Time derivative.** Trivial: $\partial _t(nC)$.

**(ii) Self-propulsion term.** For the deterministic (drift) part of the motion, it can be noted that, because ${\textbf {v}}'-{\textbf {v}}=v_0(\hat{{\textbf {u}}}'-\hat{{\textbf {u}}})$ and $\hat{{\textbf {u}}}=(1,0)$,

A17 $$\begin{aligned}&\iint \cos \psi \,({\textbf {v}}'-{\textbf {v}})\!\cdot \!\nabla _{\!r}P\,d\varphi \,d\varphi ' =v_0\,\partial _r(nC). \end{aligned}$$

However, since it restricts to drift alone, the calculation above keeps only the first so-called Kramers-Moyal coefficient of the term related to self-propulation, so it captures the ballistic drift of the mean separation but omits the higher-order moments that generate effective diffusion after the headings decorrelate. Kramers-Moyal theory (Kramers 1940; Moyal 1949; Cercignani 1969) expresses the infinitesimal propagator of any Markov process as an infinite series of differential operators. The first coefficient gives drift, and the second gives diffusion. By retaining the second coefficient in Step 4 we recover the radial and angular Laplacians that encode the random-walk broadening of the particle pair—physics that would be missed if one stopped at the drift level alone. In Step 4 we therefore revisit exactly the same term, rewrite it as a divergence of a flux, and carry out a systematic Kramers-Moyal expansion.

**(iii) Drift operators** $\mathcal {L}_{\hat{{\textbf {u}}}}$ **and** $\mathcal {L}_{\hat{{\textbf {u}}}'}$ **.** In $\mathcal {L}_{{\textbf {v}}}$ two distinct contributions appear:

1. the single-particle angular diffusion $-\nabla _{\!v}\!\cdot \!\bigl [S({\textbf {v}}){\textbf {v}}\,P\bigr ]$, which, once restricted to the fixed-speed $|{\textbf {v}}|=v_0$ (see note at the start of step **(iv)**) reduces to $\gamma \,\partial _\varphi ^2 P$ and therefore produces isotropic relaxation of each heading;
2. the drift $-\nabla _{\!v}\!\cdot \!\bigl [\int f_{\text {ar}}(r, \phi ({\textbf {v}}, {\textbf {r}})) \hat{{\textbf {r}}} \, P({\textbf {r}}, {\textbf {v}}, {\textbf {v}}', t) \, d{\textbf {r}} d{\textbf {v}}'\bigr ]$, which does not simply relax orientations but rather biases them according to the attraction-repulsion kernel.

Here we will first discuss part 1; part 2 is grouped with the explicit source term and handled later in step (v). Keeping only the single-particle part responsible for angular relaxation, one has

A18 $$\begin{aligned} (\mathcal {L}_{\hat{{\textbf {u}}}}+\mathcal {L}_{\hat{{\textbf {u}}}'})P =\gamma \bigl (\partial _{\varphi }^2+\partial _{\varphi '}^2\bigr )P. \end{aligned}$$

Using $\partial _{\varphi }^2\cos \psi =\partial _{\varphi '}^2\cos \psi =-\cos \psi$ and Equation (A16) gives

A19 $$\begin{aligned} -2\gamma (nC). \end{aligned}$$

**(iv) Velocity-space diffusion.** Because the self-propulsion dynamics quickly squeezes the speed distribution around the preferred magnitude $v_0$, we condition all probability densities on the fixed speed $|{\textbf {v}}|=v_0$ by inserting $\delta (v-v_0)$ in every phase-space integral. Acting on such shell-restricted functions removes the radial part of the velocity Laplacian,

$$ \Delta _{\!v} =\partial _v^2+\frac{1}{v}\partial _v+\frac{1}{v^2}\partial _\varphi ^2 \;\;\longrightarrow \;\; \frac{1}{v_0^{2}}\partial _\varphi ^2, $$

and analogously for the primed variables. Therefore, the operator projects at fixed speed as

$$\begin{aligned} \frac{\sigma ^2}{2}(\Delta _{\!v}+\Delta _{\!v'})\;\;\longrightarrow \;\;\frac{\sigma ^2}{v_0^2}(\partial _{\varphi }^2+\partial _{\varphi '}^2). \end{aligned}$$

Its contribution is therefore identical to (iii) upon replacing $\gamma \mapsto \sigma ^2/(2v_0^2)$; hence the angular damping rate is $\gamma =\sigma ^2/(2v_0^2)$.

**(v) Source from attraction-repulsion.** All residual terms stemming from $f_{\textrm{ar}}$ assemble into the signed source density

A20 $$\begin{aligned} S_{\textrm{ar}}(r,\theta ,t)=\rho \,g(r,\theta ,t)\,f_{\textrm{ar}}(r,\theta ). \end{aligned}$$

### Step 3: Collecting (i)-(v)

Putting pieces together yields

A21 $$\begin{aligned} \partial _t(nC)&=-v_0\partial _r(nC)-2\gamma (nC)+S_{\textrm{ar}} +\text {(to\,be\,completed\,by\,Step 4)}, \end{aligned}$$

where we have momentarily left out the spatial-diffusion pieces that enter in the next step.

### Step 4: Conservative Self-Propulsion and Diffusion Coefficients

Write the flux in divergence form

A22 $$\begin{aligned} \mathcal {T}[P]:=({\textbf {v}}'-{\textbf {v}})\!\cdot \!\nabla _{\!r}P =\nabla _{\!r}\!\cdot \bigl [({\textbf {v}}'-{\textbf {v}})P\bigr ]. \end{aligned}$$

In Step 2 (ii) we treated the self-propulsion term $({\textbf {v}}'-{\textbf {v}})\!\cdot \nabla _{\!r}P$ directly, obtaining the leading drift contribution $-v_{0}\,\partial _{r}(nC)$. That calculation kept only the first member of the Kramers-Moyal expansion of the relative-position increment. Here, we rewrite exactly the same term in a conservative form, $\nabla _{\!r}\!\cdot [({\textbf {v}}'-{\textbf {v}})P]$, and performs a systematic small-time expansion of the associated flux, $J_{r}$. In this section, it will be shown that,

**4 (ii)**: recovers the same drift $-v_{0}\partial _{r}C$, confirming the Step 2 result but not adding a new term;
**4 (iii-iv)**: keeps the second Kramers-Moyal contribution, proportional to $\langle (\Delta r)^{2}\rangle /(2\Delta t)$, which yields the diffusive operator $D_{r}(\partial _{r}^{2}+r^{-1}\partial _{r})(nC)$ together with its angular analogue.

**4(i) Radial component of the flux.** Because $({\textbf {v}}'-{\textbf {v}})$ is symmetric in $\psi \!\leftrightarrow \!-\psi$ and $\cos \psi$ is even, only the radial component $J_r$ survives,

A23 $$\begin{aligned} J_r&:=\iint \cos \psi \,({\textbf {v}}'-{\textbf {v}})\!\cdot \!\hat{{\textbf {e}}}_r\,P\,d\varphi \,d\varphi '\end{aligned}$$

A24 $$\begin{aligned}&= -\tfrac{1}{2} v_0\,n(r,\theta ,t)\,C(r,\theta ,t). \end{aligned}$$

Note that the factor $-\tfrac{1}{2}$ comes from $\int _0^{2\pi }\cos \psi (\cos \psi -1)d\psi =-\pi$.

**4(ii) Drift term.** Substituting $J_r$ into $-(1/n)\partial _rJ_r$ reproduces the drift $-v_0\partial _rC$ obtained earlier, confirming Equation (A17) by an alternative route.

**4(iii) Diffusive correction.** Write the radial increment over a short interval $\Delta t$ as

$$ \Delta r =({\textbf {v}}'-{\textbf {v}})\!\cdot \hat{{\textbf {e}}}_{r}\,\Delta t +\sqrt{2D_{0}\,\Delta t}\,\eta _{r}, \qquad D_{0}:=\sigma ^{2}/2,\quad \langle \eta _{r}^{2}\rangle =1 . $$

The first term is ballistic; the second represents Gaussian translational noise. The second Kramers-Moyal coefficient of the self-propulsion term is $\tfrac{1}{2}\,d\langle (\Delta r)^{2}\rangle /dt$. This coefficient allows to integrate over a time window that is long compared with the angular persistence. To choose such a typical time scale, $\tau _{p}=1/\gamma$, observe the following. Recall that for an isolated self-propelled particle the heading obeys the angular Fokker-Planck equation (see Equation A18), which is given by $\partial _t P(\varphi ,t)=\gamma \,\partial ^2_{\varphi }P$, so that the unit-vector autocorrelation decays exponentially,

$$ \langle \hat{{\textbf {u}}}(t)\!\cdot \!\hat{{\textbf {u}}}(0)\rangle = e^{-\gamma t}. $$

The characteristic decay time, $\tau _{p}=1/\gamma$, is therefore the persistence time of a single heading. For time intervals $\Delta t\ll \tau _{p}$ the relative displacement is ballistic, while for $\Delta t\gg \tau _{p}$ the headings have decorrelated and the ballistic piece averages to zero. Therefore, coarse graining on a time scale $\Delta t \sim \tau _p$ gives the effective radial diffusivity,

By standard Kramers-Moyal theory we then obtain the radial Laplacian, which is given by $D_{r}\,(\partial _{r}^{2}+r^{-1}\partial _{r})(nC)$. The same construction applied to the angular increment yields the angular term, $(D_{\theta }/r^{2})\partial _{\theta }^{2}(nC)$, with $D_{\theta }=\sigma ^{2}/2$.

**4(iv) Divergence in polar coordinates.** Since the flux is radial, $\nabla _{\!r}\!\cdot {\textbf {J}}=\partial _rJ_r+r^{-1}J_r$. Replacing $J_r=-D_r\partial _r(nC)$ gives

A25 $$\begin{aligned} \nabla _{\!r}\!\cdot {\textbf {J}}=D_r\bigl (\partial _r^2+r^{-1}\partial _r\bigr )(nC). \end{aligned}$$

Identical reasoning for the angular increment delivers the angular term $(D_\theta /r^2)\partial _\theta ^2(nC)$ with $D_\theta =\sigma ^2/2$.

**Putting all pieces together.** Combining the results of 4(ii) – 4(iv), the entire self-propulsion part of the Fokker-Planck operator acts on $nC$ as

$$ -\,v_{0}\,\partial _{r}(nC) \;+\; D_{r}\!\bigl (\partial _{r}^{2}+r^{-1}\partial _{r}\bigr )(nC) \;+\; \frac{D_{\theta }}{r^{2}}\partial _{\theta }^{2}(nC). $$

Here, the first term reproduces the ballistic drift already obtained in Step 2(ii) and the second and third terms are the diffusive corrections generated by the second Kramers-Moyal coefficient after coarse-graining over the persistence time $\tau _{p}=1/\gamma$. Taken together, these three operators exhaust the influence of self-propulsion. Including the diffusion pieces into (A21) produces

A26 $$\begin{aligned} \partial _t(nC)= -v_0\partial _r(nC) +D_r(\partial _r^2+r^{-1}\partial _r)(nC) +\frac{D_\theta }{r^2}\partial _\theta ^2(nC) -2\gamma (nC) +S_{\textrm{ar}}. \end{aligned}$$

## Appendix C: Density-Division: Exact Algebra and Controlled Truncation

Write $n=\rho g$ and let $\mathcal {L}$ denote any linear differential operator acting on *nC*. Then

A27 $$\begin{aligned} \frac{1}{n}\,\mathcal {L}(nC)=\mathcal {L} C + (\mathcal {L}\ln n)C +\sum _{\mu }(\partial _\mu C)(\partial _\mu \ln n)+C\,\partial _\mu ^2\ln n+\dots \,. \end{aligned}$$

Applying this identity to every term in (A26) gives an exact equation for *C*. Introduce $L_C$ (variation scale of *C*) and $L_n$ (scale of *n*) and define $\varepsilon =L_C/L_n\ll 1$. Counting gradients shows that all terms containing any $\partial _\mu \ln n$ are at least $\mathcal {O}(\varepsilon )$ relative to the retained ones. Discarding them yields, to leading order in $\varepsilon$,

A28 $$\begin{aligned} \partial _tC=-v_0\partial _rC+D_r(\partial _r^2+r^{-1}\partial _r)C +\frac{D_\theta }{r^2}\partial _\theta ^2C-2\gamma C+\frac{S_{\textrm{ar}}}{n}. \end{aligned}$$

Higher-order corrections are obtained by keeping $\mathcal {O}(\varepsilon )$ terms.

## Appendix D: Symbol Table

**Table:**

Symbol	Meaning/definition
$\rho$	Bulk particle number density.
$v_0$	Mean self-propulsion speed (assumed constant).
$\sigma$	Translational noise amplitude.
$\gamma =\sigma ^2/(2v_0^2)$	Single-particle angular decorrelation rate.
$D_r=(v_0^2+\gamma \sigma ^2)/2$	Radial diffusivity of the pair separation.
$D_\theta =\sigma ^2/2$	Angular diffusivity of the separation vector.
$S_{\textrm{ar}}$	Source term from the attraction-repulsion kernel.
$g=n/\rho$	Pair distribution function.

## Appendix E: Velocity Alignment Contributions

Starting from the pair Fokker-Planck equation, the alignment drift augments the operator $L_v$ by

A29 $$\begin{aligned} L^{al}_v[P] \;=\; -\nabla _v\!\cdot \!\Bigg [ \int f_{al}(r,\varphi (v,r))\,(I-\hat{u}\hat{u}^\top )\,\hat{u}'\;P(r,v,v',t)\,dr\,dv' \Bigg ], \end{aligned}$$

and analogously for $L_{v'}$. Project to the speed shell as before is immediate by replacing $v\mapsto v_0\hat{u}$, $\,v'\mapsto v_0\hat{u}'$, and integrating over $v,v'$ against $\delta (v-v_0)\delta (v'-v_0)\,v\,v'$. Defining *n*, *g*, *C* as in Appendix A. Multiplying the *P*-equation by $\hat{u}\!\cdot \!\hat{u}'$, integrating over the angular variables, and cataloguing terms exactly as before allows us to identify:

- **(i) Time derivative:** $\partial _t(nC)$ remains unchanged.
- **(ii) Self-propulsion:** $-v_0\partial _r(nC)$ (ballistic drift) and, after Kramers–Moyal coarse-graining over $\tau _p=1/\gamma$, the radial and angular Laplacians acting on *nC* with coefficients $D_r$ and $D_\theta$ are unchanged
- **(iii) Single-particle angular relaxation:** $-2\gamma (nC)$ unchanged
- **(iv) Interaction sources:** one from the attraction-repulsion kernel and one from alignment:
  $$ S_{ar}(r,\theta ,t)=\rho \,g(r,\theta ,t)\,f_{ar}(r,\theta ) \quad \text {and} \quad S_{al}(r,\theta ,t)=\rho \,g(r,\theta ,t)\,f_{al}(r,\theta ), $$
  obtained by inserting the shell projection into the interaction drifts in $L_v$ and $L_{v'}$ and using the same angular identities as in Step 2 of Appendix A.

Collecting pieces gives the *nC*-equation with alignment,

$$ \partial _t(nC)= -v_0\partial _r(nC)+D_r(\partial _r^2+r^{-1}\partial _r)(nC)+\frac{D_\theta }{r^2}\partial _\theta ^2(nC)-2\gamma (nC)+S_{ar}+S_{al}. $$

Applying the density-division identity, discarding $O(\varepsilon )$ gradient terms as in Appendix C, and dividing by $n=\rho g$ yields immediately Equation (36).

Now, writing the alignment kernel in harmonics,

$$ f_{al}(r,\theta )=\sum _{\ell \ge 0}A_\ell (r)\cos (\ell \theta ),\qquad \widehat{A}_\ell (k)=2\pi \int _0^\infty r\,A_\ell (r)J_\ell (kr)\,dr, $$

the first harmonic ($\ell =1$) contributes only to the dipole instability by producing a diagonal shift in that block. Projecting the interaction matrix onto $p=\pm 1$ (the same step used to obtain $\alpha (k)$ in the attraction-repulsion only case) yields the additional shift,

$$ \alpha _{al,1}(k)=\frac{\rho _0 k^2}{2}\,\widehat{A}_1(k). $$

Therefore, the dipole eigenvalue becomes Equation (37) with $\alpha _{ar,1}$ and $\alpha _{al,1}$ added. In contrast, the side block $M_2(\lambda )$ does not involve $p=\pm 1$, so the presence of a dipolar alignment harmonic leaves the side eigenvalues unchanged. In the large-Péclet simplification, the isotropic condition that $\rho _0\widehat{F}(k)>\sigma ^2/2$ remains unchanged. The dipole branch becomes unstable when $\lambda _{\textrm{dip}}(k)$ in Equation (37) becomes positive, *i.e.*, when the combined shift $\alpha _{ar,1}(k)+\alpha _{al,1}(k)$ reduces the effective angular damping in the dipole block sufficiently for the term $\beta ^2/(\lambda _r^{(0)}+D_\theta )$ to push the eigenvalue across 0. A sufficient condition for a suppressed dipole pattern is therefore given by the band $D_\theta<\lambda _r^{(0)}(k)<D_\theta -(\alpha _{ar,1}(k)+\alpha _{al,1}(k))$.
